# Erythroferrone and hepcidin levels in children with iron deficiency anemia

**DOI:** 10.1186/s12887-024-04594-5

**Published:** 2024-04-04

**Authors:** Ramazan Dulkadir, Gamze Turna Saltoğlu, Ali Güneş

**Affiliations:** Ahi Evran Training and Research Hospital Pediatrics Clinic Bagbasi, Kırşehir Ahi Evran University, Kirsehir, Turkey

**Keywords:** Iron, anemia, Hepcidin, Erythroferrone, Children, Correlation

## Abstract

**Background:**

Iron deficiency anemia remains a significant public health issue in developing countries. The regulation of iron metabolism is primarily controlled by hepcidin, a key regulatory protein. During erythropoiesis, erythroferrone (ERFE), a hormone produced by erythroblasts in response to erythropoietin (EPO) synthesis, mediates the suppression of hepcidin. In this study, it was aimed to determine the correlation between erythroferrone (ERFE) and hepcidin levels in children with iron deficiency anemia.

**Methods:**

This is a case-control study conducted at Kırşehir Ahi Evran University Training and Research Hospital Pediatrics Clinic between 1 and 31 September 2020. The study included 26 healthy children and 26 children with iron deficiency anemia. In order to evaluate iron status,whole blood count, serum iron, total iron binding capacity (TIBC), and ferritin levels were analyzed. The study measured the levels of hepcidin and erythroferrone in the serum of children diagnosed with iron deficiency before and after one month of iron treatment, as well as in a control group, using the ELISA method. Correlation between whole blood count, initial ferritin, hepcidin, ERFE and ferritin in the iron deficiency group was evaluated.

**Results:**

Compared with healthy controls, the iron-deficient group had significantly lower haemoglobin (*p* < 0.001), MCV (*p* = 0.001), MCH (*p* < 0.001), MCHC (*p* < 0.001), iron (*p* < 0.001), ferritin (*p* < 0.001) and hepcidin (*p* = 0.001). Ferritin and hepcidin levels increased while erythroferrone levels remained unchanged after iron deficiency treatment. There was no correlation between hepcidin and ferritin levels in treatment group.

**Conclusions:**

The study found a strong and positive correlation between ferritin and hepcidin levels in iron-deficient children, but not between ERFE levels, suggesting that hepcidin is largely regulated by iron deposition levels. In addition, there was an increase in ferritin and hepcidin levels after iron treatment. The study found no significant difference in erythroferrone levels between the iron-deficient group and the control group. It is thought that this may be due to the short duration of iron treatment given to the patients with iron deficiency anemia included in the study.

## Introduction

Anemia is defined as the decrease of hemoglobin (Hb), hematocrit (Htc), and erythrocyte count (RBC) below the value accepted as normal for a certain age and gender by two standard deviation value [1]. The issue of anemia, which has preserved its importance until today, continues to be a significant health problem especially in developing countries (2–3). It is estimated that at least 30% of the world population are affected by anemia. This rate is between 4 and 20% in developed countries and 70–80% in developing countries [4]. In studies conducted in our country covering different age groups, the prevalence of iron deficiency anemia (IDA) was found to be between 15 and 30%. IDA prevalence that reaches up to 50% especially in infancy has been reported in studies [5].

Iron deficiency is widespread all over the world, and it is especially a public health problem that threatens life. The most common causes of iron deficiency in children are iron-poor nutrition or food intake deficiency, absorption disorders, feeding with cow milk before a certain age, situations such as parasitosis, obesity, maternal deficiency, and prematurity [6].

One of the most important consequences of iron deficiency observed in childhood is mental and motor development disorder (7–8).

Iron is a crucial metal for all cells. Both iron deficiency and iron overload can have harmful effects on the body [9]. The majority of the iron in the body is bound to hemoglobin [9]. A significant portion of the body’s iron is stored in hepatocytes as ferritin. Hepcidin, mainly expressed in hepatic tissue, is one of the primary regulators of iron homeostasis. It controls iron absorption and distribution in various tissues [10]. Hepcidin controls iron homeostasis by reducing iron absorption from nutrients in the duodenum, stopping iron release from macrophages in the circulation, and controlling the transportation of iron deposits in hepatocytes [10]. When the plasma iron concentration increases, hepcidin mRNA transcription in hepatic tissues increases [10]. Hepcidin regulates iron balance by reducing the amount of iron in circulation. It achieves this by preventing iron absorption from enterocytes and iron release from macrophages. Total deficiency of hepcidin leads to an increase in iron absorption, which can cause juvenile hemochromatosis, a disorder characterized by excessive iron accumulation in the body [11]. Conversely, excessive release of hepcidin results in severe iron deficiency anemia (IDA) as iron absorption is reduced, even though a sufficient amount of iron is taken into the body [12]. Erythroferrone (ERFE) is secreted in the bone marrow and spleen by erythroid precursors as a response to erythropoietin (EPO) stimulation [13]. ERFE increases hepcidin suppression during increased erythropoietic activity, and thus it increases usability of iron for new erythrocyte synthesis [14]. Elevated iron levels and inflammation lead to an increase in hepcidin transcription. As a result, the synthesis of hepcidin is suppressed by erythropoietic activity. Under the influence of EPO, ERFE secretion is activated in erythroblasts, which impairs the production of hepcidin, facilitating the uptake of iron from the diet and the release of iron from macrophage stores. Erythroferrone has a beneficial role in infection-related anemias by suppressing hepcidin and increasing iron release from cellular iron deposits [14].

In the study, it was aimed to investigate the correlation between ERFE and hepcidin levels in patients with iron deficiency anemia and the parameters in iron deficiency anemia, and by looking into ERFE and hepcidin levels, to determine whether the factors that will affect ERFE and hepcidin levels will make an additional contribution to the diagnosis and treatment of iron deficiency anemia.

## Materials and methods

### Study population

This is a case-control study conducted at Kırşehir Ahi Evran University Training and Research Hospital Pediatrics Clinic between 1st September 2020 and 31st September 2020. The study included 26 healthy children and 26 children with iron deficiency anemia. Normal hemoglobin and hematocrit lower limits according to age and gender and red cell indices based on normal age and gender determined by the World Health Organization were used for the diagnosis of anemia. All the procedures were followed in accordance with the Declaration of Helsinki. In order to evaluate iron status, serum iron, total iron binding capacity (TIBC), and ferritin values were analyzed.

The study was conducted with three groups.Group 1 consisted of 26 cases diagnosed with iron deficiency anemia who have not yet commenced treatment. The inclusion criteria for this group require that patients have no chronic physiological diseases, haemoglobinopathies or infections that could affect ferritin levels.The group included patients with microcytic anaemia and iron deficiency based on iron, iron binding capacity, and ferritin levels. To diagnose IDA, we used the World Health Organization’s normal age- and sex-specific lower limits for haemoglobin and haematocrit, as well as their normal age- and sex-specific erythrocyte indices, to diagnose the anaemia cases in group 1.

Group 2 consisted of the same patients as in group 1. The patients in this group were treated with 6 mg/kg of ferrous iron preparations for one month. (*n* = 26)

Group 3: The study sample consisted of children who had undergone a healthy child examination at our hospital and who did not have any chronic complaints(Control group). Specifically, the sample included children between the ages of 0 and 18 years with no health problems. We excluded children with chronic diseases and/or medical conditions that could affect iron homeostasis, resulting in a final sample size of *n* = 26.

In order to reduce bias in the selection of patients, pre-defined, comprehensive criteria were used to identify individuals for inclusion in the study. These criteria were set in accordance with the main aims and hypotheses of the research. In addition, methods such as random assignment or the creation of balanced groups were used to minimise potential bias between groups. Each step in this selection process was carefully planned and implemented to increase the reliability and internal validity of the research.

The study protocol was approved by Kırşehir Ani Evran University Medical Faculty Clinical Research Ethics Committee (Decision no: 16/119 Date: 2020). Informed consents were obtained from the patients and parents if the child is older than 12 years and from only the parents if the patient is younger than 12 years.

The study was supported as Kırşehir Ani Evran University Scientific Research Project (Project no: Medicine A4-20 006).

### Laboratory measurements

Blood samples were taken from all cases into plain serum tubes and EDTA (ethylenediaminetetraacetic acid) tubes following one night fasting. The blood samples were centrifuged at 200 x g for 10 min. The serum samples were separated into aliquots and were kept at -80 ^o^C until study day for hepcidin and erythroferrone measurements. The hematological statuses of the cases were determined by using hematology analyzer (Sysmex, XN-1000). Iron, iron binding, and ferritin measurements were made in autoanalyzers (AU 5800; Beckman Coulter, USA; DXI 800; Beckman Coulter, USA, respectively).

Serum hepcidin and erythroferrone levels were determined by using Human Hepcidin ELISA kit (Elabscience Biotechnology Inc., USA) and Human Erythroferrone ELISA kit (USCN Life Science Inc., Wuhan, China ) in line with the instructions of the manufacturers.

### Statistical analysis

In the analysis of the data, SPPS 25 (IBM Corp. Released 2017. IBM SPSS Statistics for Windows, Version 25.0. Armonk, NY: IBM Corp.) package software was used. The variables were summarized as mean ± standard deviation, median (Minimum-Maximum), percentage, and frequency. The variables were evaluated after the normality and homogeneity assumption controls were made (Shapiro-Wilk and Levene Test). In the analysis of the data, Independent Two Groups t test (student’s t test) was used in the comparison of two groups, and when the assumptions were not met, Mann-Whitney U test was employed. For the comparison of 3 groups and more, One-Way Analysis of Variance (ANOVA) and Tukey HSD test, one of the multiple comparison tests, were used, and when the assumptions were not met, Kruskal Wallis and Bonferroni-Dunn test, one of the multiple comparison tests, were employed. The relationship between two continuous variables was analyzed with Pearson Correlation Coefficient, and when the parametric test assumption was not met, Spearman Correlation Coefficient was used. The boundary value for iron value was determined through ROC analysis. *p* < 0.05 and *p* < 0.001 were accepted as significance levels for the tests.

## Result

52 children were included in the study. Of the patients diagnosed with iron deficiency anemia, 57.7% were girls (n:15) and 42.3% were boys (n:11), while 65.3% of Group 3 were girls (n:17) and 34.7% were boys (n:9). The mean age of groups 1 and 2 was 7.62 ± 3.77 years, while the mean age of group 3 was 9.46 ± 3.9 years. No significant difference was found between the groups in terms of gender and age (*p* = 0.430,*p* = 0.122). (Table 1).

**Table 1:** Demographic and laboratory characteristics of the study groups before the study

	Group 3 (*n* = 26)	Group 1 (*n* = 26)	*P* value
Age	9.46 ± 3.93	7.62 ± 3.77	0.090
Gender			
Boy	9 (34.7%)	11 (42.3%)	0.430
Girl	17 (65.3%)	15 (57.7%)	
Leukocyte count (mm^3^)	7.53 ± 1.76	8.21 ± 2.15	0.292
Hemoglobin(g/dL)	13.87 ± 0.95	12.45 ± 0.78	**< 0.001**
Hematocrit (%)	39.5 ± 2.68	38.63 ± 3.25	0.304
MCV (fl.)	79.68 ± 3.91	75.36 ± 4.8	**0.001**
MCH (pg)	28.03 ± 1.78	25.45 ± 1.96	**< 0.001**
MCHC (g/dl)	35.18 ± 1.19	33.75 ± 1.37	**< 0.001**
Platelet count (mm^3^)	313.6 ± 72.3	335.9 ± 67.7	0.260
RDW	12.43 ± 0.72	13.38 ± 1.06	**0.001**
Neutrophil %	45.63 ± 11.79	42.58 ± 16.67	0.458
Leukocyte %	43.02 ± 11.39	43.88 ± 17.93	0.955
Monocyte %	7.63 ± 1.67	7.26 ± 2.27	0.521
Eosinophil %	2.47 ± 2.7	2.06 ± 2.81	0.599
Iron (µg/mL)	105.62 ± 33.44	43.38 ± 12.16	**< 0.001**
Iron binding capacity (µg/mL)	395 ± 44.78	416.77 ± 56.69	0.135
Ferritin(ng/mL)	30.62 ± 12.92	15.16 ± 9.13	**< 0.001**
Hepcidin (pg/mL)	4358.3 ± 840.3	2600.9 ± 1588.1	**< 0.001**
Erythroferrone (ng/mL)	0.137 ± 0.003	0.143 ± 0.020	0.133

MCV: Mean erythrocyte value, MCH: Mean erythrocyte hemoglobin, MCHC: Mean erythrocyte hemoglobin concentration, RDW: Erythrocyte distribution width

In comparison to group 3, hemoglobin (*p* < 0.001), MCV (*p* = 0.001), MCH (*p* < 0.001), MCHC (*p* < 0.001), iron (*p* < 0.001), ferritin (*p* < 0.001) and hepcidin (*p* = 0.001) were significantly lower in group 1. In addition, RDW was significantly higher in group 1 compared to group 3 (*p* = 0.001) (Table 1). Comparing group 1 and group 2, significant increases were observed in erythrocyte count (*p* = 0.034), hemoglobin, hematocrit, MCV (*p* < 0.001 each), MCHC (*p* = 0002), iron (*p* < 0.001), ferritin (*p* = 0.015) and hepcidin (*p* < 0.001). At the end of treatment, iron binding capacity showed a significant decrease (*p* = 0.004) (Table 2).

**Table 2:** Comparison of group 1 and group 2

	Group 1	Group 2	*P* value
Leukocyte count (mm^3^)	8.21 ± 2.15	7.33 ± 1.37	0.108
Hemoglobin(g/dL)	12.46 ± 0.78	13.49 ± 0.95	**< 0.001**
Hematocrit (%)	38.63 ± 3.25	40.79 ± 2.47	**< 0.001**
MCV (fl.)	75.36 ± 4.8	78.49 ± 4.38	**< 0.001**
MCH (pg)	25.45 ± 1.96	27.15 ± 6.66	0.158
MCHC (g/dl)	33.75 ± 1.37	33.09 ± 1.12	**0.002**
Platelet count (mm^3^)	335.9 ± 67.7	348 ± 73.5	0.323
RDW	13.38 ± 1.06	13.71 ± 1.33	0.248
Neutrophil %	42.72 ± 17.02	42.5 ± 12.32	0.952
Lymphocyte %	43.56 ± 18.25	44.4 ± 11.44	0.800
Monocyte %	7.26 ± 2.32	7.54 ± 2.28	0.546
Eosinophil %	2.08 ± 2.87	2.36 ± 2.39	0.510
Iron r(µg/mL)	43.38 ± 12.16	66.92 ± 23.17	**< 0.001**
Iron binding capacity (µg/mL)	416.8 ± 56.7	386.5 ± 35.6	**0.004**
Ferritin (ng/mL)	15.16 ± 9.13	19.24 ± 9.32	**0.015**
Hepcidin (pg/mL)	2600.9 ± 1588.1	3820.5 ± 1457.2	**< 0.001**
Erythroferrone (ng/mL)	0.143 ± 0.019	0.139 ± 0.007	0.157

MCV: Mean erythrocyte volume, MCH: Mean erythrocyte hemoglobin, MCHC: Mean erythrocyte hemoglobin concentration, RDW: Erythrocyte distribution width

**Fig. 1:**
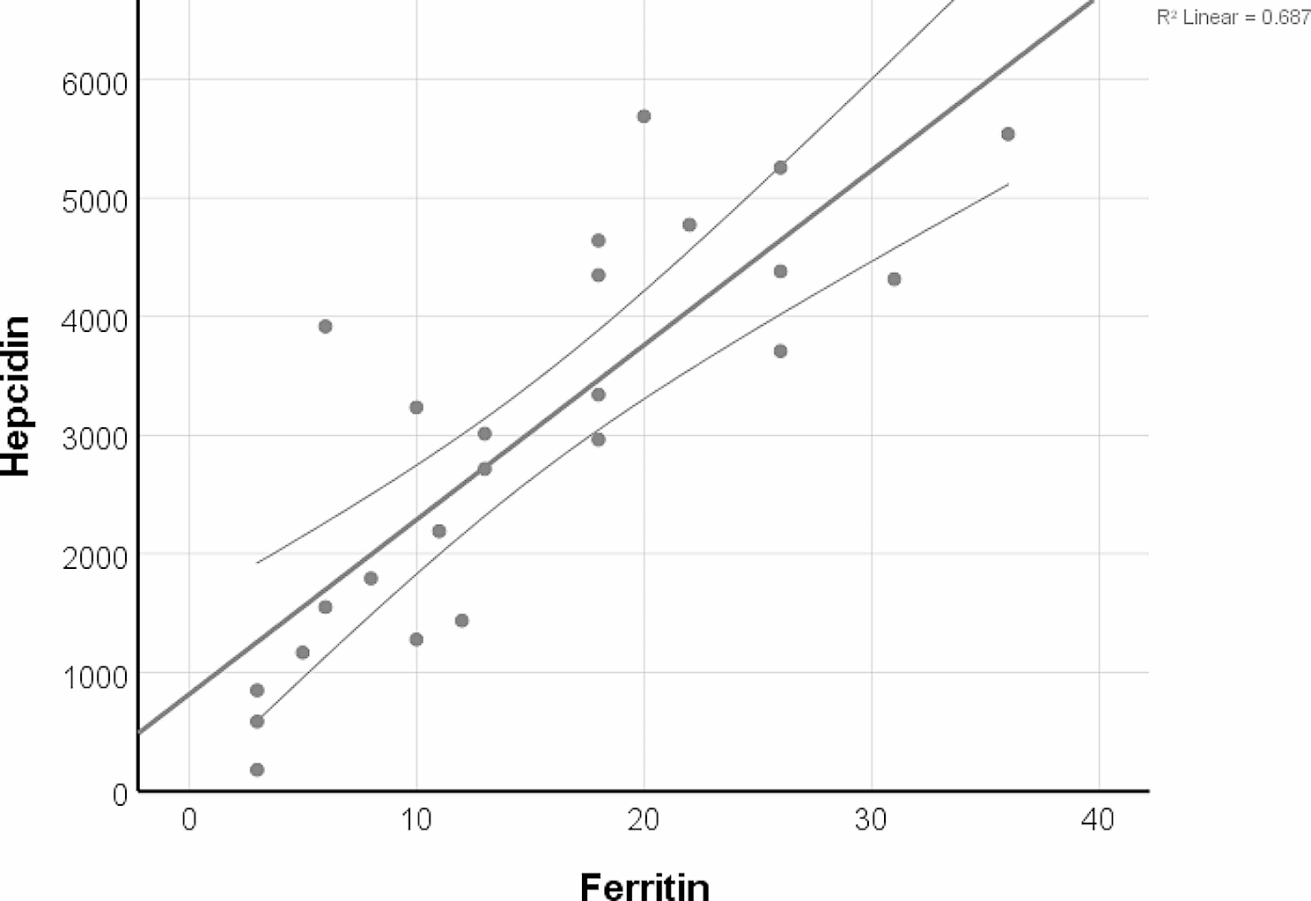
Correlation between initial hepcidin and ferritin in the iron deficiency group (95% confidence interval)

In group 1, initial hepcidin and ferritin levels were significantly and positively correlated (*r* = 0.829, *p* < 0.001) (Fig. 1).

In group 2, however, erythroferrone levels showed no correlations, but hepcidin levels showed moderate or weak correlations with neutrophil count (*r* = -0.429, *p* = 0.037), eosinophil count (*r* = -0.447, *p* = 0.029) and iron levels (*r* = 0.594, *p* = 0.002). In addition, there was no correlation between the levels of hepcidin and ferritin in group 2.

## Discussion

Iron deficiency is a widespread public health problem in our country and around the world. It is a major threat to the paediatric age group in particular. It is believed that the assessment of ERFE and hepcidin levels in patients with iron deficiency anemia will provide guidance for new treatment methods in the future.

In this study, as expected, hemoglobin, MCVs, MCHs, MCHCs and RDWs, iron, ferritin and hepcidin levels were lower in the iron-deficient group.

As an essential regulator of systemic iron balance, hepcidin transcription in the liver is controlled by a complex interplay of signals including overt inflammation, iron status and erythropoietic triggering [15]. Iron in the circulation and tissues increases hepcidin levels to inhibit further iron uptake, and erythropoietic activity suppresses hepcidin to increase the availability of iron for erythropoiesis [16].

In iron deficiency, hepcidin decreases and iron absorption increases, leading to iron release from depots [17]. In a study by Semercioglu et al., hepcidin levels were found to be significantly lower in children with iron deficiency anemia than in children without iron deficiency anemia [18]. In parallel with this study and the literature, the present study found that hepcidin levels were significantly lower in the iron deficiency group compared to the control group (Group 3).

Hepcidin also acts as a mediator in host defence. During infection, cytokines (especially interleukin) increase and hepcidin levels rise very rapidly, leading to an acute decrease in plasma iron concentration [17]. Decreased plasma and non-cellular iron concentrations limit the iron supply required for infectious agents [17]. Inflammatory anemia is characterised by high hepcidin concentrations stimulated by inflammatory cytokines [17]. A study of children with inflammatory bowel disease found a positive correlation between hepcidin and ferritin [19]. Similarly, the present study found a strong correlation between ferritin and hepcidin, but no correlation with other laboratory parameters. This may suggest that hepcidin is largely regulated by iron deposition levels.

In this study, it was found that a positive correlation between initial hepcidin levels and initial ferritin levels (*r* = 0.829, *p* < 0.001). On the other hand, erythroferrone levels did not display any correlations.

In cases of recurrent blood loss, hepcidin is suppressed by erythroferrone and this allows more iron absorption which is necessary for the recovery of anemia [17]. Ganz et al. found a negative correlation between ERFE and hepcidin concentrations [20]. On the other hand, another study found no correlation was found between plasma ERFE and hepcidin concentrations [21]. Hepcidin suppression during erythropoiesis is mediated by ERFE [22]. Therefore, we expected that decreased hepcidin levels would be accompanied by increased ERFE. In the present study, no correlation was determined between ERFE and hepcidin concentrations. Similar to this study, it was found in other studies that ERFE is most likely not the only factor in the suppression of hepcidin expression [23].

In another study, ERFE concentrations were found to be higher in iron-deficient individuals compared with ERFE concentrations in controls (group 3) [24]. In this study, a negative correlation was found between ERFE and ferritin concentrations. These researchers were unable to demonstrate a relationship between hepcidin and ERFE as they did not measure hepcidin levels [24]. In the present study, no correlation was found between hepcidin and erythroferrone, nor between ferritin and erythroferrone.

In a study similar to the present study by Xu LH et al., when serum hepcidin levels were examined between women who gave birth to children with IDA and women who gave birth to children without IDA, it was found that hepcidin decreased significantly in the iron-deficient group, but there was no significant difference in erythroferrone levels between the two groups [25]. Studies have reported that iron deposition reaches its maximum level 2–3 months after the start of treatment [26]. No significant change in erythroferrone levels was seen in this study. It is thought that this may be due to the short duration of iron treatment given to the patients with iron deficiency anemia included in the study. If the suppressive role and mechanism of action of ERFE on hepcidin can be clarified as a result of future studies, antibodies specific to ERFE can be produced. More detailed studies are needed to elucidate the role of ERFE in iron metabolism.

In conclusion, the study found that serum hepcidin levels decrease in iron deficiency anemia, provided there are no inflammatory processes. The positive and strong correlation found between hepsidin and ferritin suggests that both are effective in iron balance. The lack of a significant difference in serum erythroferrone levels between the control group (group 3) and the iron deficiency group suggests that erythroferrone may not play a role in regulating iron metabolism in children with iron deficiency anemia.

### Limitations

This study did not complete the 3-month treatment period, which is the time necessary for the replenishment of iron stores for the treatment of iron deficiency. In future trials, the 3-month treatment period should be completed. In addition, an increase in the number of people in the study may have a greater impact on the results.

## Data Availability

The datasets used and/or analysed during the current study available from the corresponding author on reasonable request.
